# Knowledge, attitudes and practice by health professionals toward medical and pharmaceutical waste management: a cross-sectional study of El-idrissi hospital, Kenitra, Morocco

**DOI:** 10.3389/frhs.2026.1756949

**Published:** 2026-02-24

**Authors:** Abdelfattah Bouchama, Miloud Chakit, Nadia Mountaj, Khadija Fritah, Amar Habsaoui

**Affiliations:** 1 Advanced Materials and Process Engineering Laboratory, Faculty of Sciences, Ibn Tofail University, Kenitra, Morocco; 2 National School of Public Health, Rabat, Morocco; 3 Informatics Research Laboratory, Faculty of Sciences, Ibn Tofail University, Kenitra, Morocco; 4 Higher Institute of Nursing and Health Techniques of Fez, Annex of Meknes, Meknes, Morocco; 5 Higher Institute of Nursing Professions and Health Techniques, Kenitra, Morocco

**Keywords:** attitudes, health professionals, Kenitra, knowledge, medical waste management, Morocco, practice

## Abstract

**Introduction:**

The safe management of medical and pharmaceutical waste (MPW) is a major challenge for preventing environmental and health risks in hospitals. In Morocco, several studies still highlight shortcomings in knowledge, practices, and risk management related to MPW. To assess the knowledge, attitudes, and practices (KAP) of healthcare staff regarding MPW management at El Idrissi Hospital in Kenitra, as well as their perception of the associated risks.

**Methods:**

A descriptive cross-sectional study was conducted among healthcare staff using an anonymous questionnaire with four sections: sociodemographic characteristics, knowledge of MPW management procedures, management attitudes, and risk perception. A total of 136 participants were included after excluding four invalid questionnaires. The data were analyzed descriptively.

**Results and Discussion:**

The majority of participants were women (67.6%). Nearly half of the staff (41.2%) were under 35 years old, with a mean age of 38 years (SD = 10.4). The mean administrative seniority was 13 years (SD = 9.2), and 47.1% had less than 10 years of experience. Staff came from various services and departments. A minority (18.8%) of them benefited from a day of awareness training on the management of medical waste (χ² = 8.64, *p* = 0.003). Meanwhile, 25% of them attended a meeting on this topic (χ² = 7.30, *p* = 0.007). Conversely, we found no significant associations (*p* > 0.05) with the media as a means of raising awareness, suggesting the need for enhanced training and improved organizational conditions.

**Conclusion:**

This study highlights persistent gaps in medical waste management at El Idrissi Hospital. Strengthening staff skills, improving internal organization, and regularly updating protocols are essential to ensure the safe management of hospital waste and reduce risks to healthcare workers, patients, and the environment.

## Introduction

Poorly managed hospital waste represents a real and persistent threat to public health (1). Its danger is not limited to its visual appearance; it originates from highly contaminated medical areas and often carries pathogenic microorganisms capable of causing serious or even fatal infections in exposed individuals. This risk concerns healthcare workers as well as those responsible for collection, not to mention populations living near sites of inappropriate disposal or treatment (2).

Indeed, all germs capable of causing human disease can be present in this waste. Therefore, its handling requires absolute vigilance. Medical personnel, particularly those involved in waste management, must apply strict protocols to limit the risk of cross-contamination. At this level, training, protective equipment, and compliance with standards are fundamental (3, 4).

Waste sorting and collection are the first and crucial steps in any safe management strategy. Each hospital department and each treatment room must have a dedicated collection system. Waste must be immediately packaged in suitable containers and then safely transported to a centralized collection point, where a hospital hygiene service is in place. This is where the role of collection agents becomes essential (3, 5).

Sharp objects, such as used needles, scalpels, or syringes, are among the most hazardous waste. They must be disposed of immediately upon use in rigid, puncture-resistant, hermetically sealed safety boxes, specially designed to prevent any risk of accidents or illicit recycling (6, 7).

The temporary storage of medical waste also meets specific standards. It must be done in large, hermetically sealed containers, protected from insects, rodents, stray animals, and unauthorized persons. When prolonged storage is necessary, these containers must be refrigerated to limit the decomposition of biological materials and microbial growth. Sealing is essential to prevent any leakage of contaminated liquids. Finally, waste must be packaged in waterproof, non-reusable bags, suitable for humidity, and hung in specific supports. In some cases, these bags are themselves inserted into plastic or metal containers to increase security. This double packaging limits risks during transport and guarantees more efficient disposal in treatment units, where they exist (8, 9).

In public hospitals, as well as in some private clinics, it is still common to find medical waste, including that classified as hazardous or infectious, collected without prior sorting and stored in a single depot, sometimes within hospital grounds. It remains piled up there for several days, without proper treatment, often within reach of scavengers, children, or stray animals. This direct exposure increases the risk of cross-contamination and reveals the shortcomings of a system that remains poorly structured (10, 11).

This waste is not harmless. They include sharp objects, equipment contaminated with bodily fluids, pharmaceutical residues, and highly toxic chemicals. Their careless handling, their transport without scientific supervision, their burial on the ground, or their mixing with household waste in landfills are all dangerous practices still observed in certain regions. Worse still, this waste is sometimes recovered in parallel circuits for reuse or fraudulent recycling, particularly syringes and needles, which represent a major risk of disease transmission (12, 13).

According to the latest report from the World Health Organization (WHO, 2026), nearly 15% of waste produced by healthcare facilities worldwide is considered hazardous, including infectious and pharmaceutical waste. In Morocco, the National Environmental Regulatory Agency (ANRE, 2025) reports that more than 60% of hospitals lack a comprehensive medical waste management system, exposing staff and patients to biological and chemical risks. This situation underscores the importance of assessing the knowledge, attitudes, and practices (KAP) of hospital staff to identify gaps and propose improvement measures tailored to the national context. A simple needlestick accident with an infected needle is enough to transmit these viruses, with respective contamination probabilities of 30% for hepatitis B, 1.8% for hepatitis C, and 0.3% for HIV.

In Morocco, healthcare facilities generate approximately 38,000 tons of medical waste each year, of which nearly 12,000 tons are considered hazardous. This high proportion considerably complicates its treatment, especially since few facilities have suitable facilities such as modern incinerators, disinfection stations, or secure collection systems. In some areas, these facilities are simply nonexistent or out of service (14).

Hospital waste management in Morocco is a growing concern among healthcare professionals, environmental stakeholders, and citizens. While hospitals' mission is to provide care, they can also, paradoxically, become sources of serious health risks when they fail to ensure the safe and controlled disposal of their medical waste. Numerous publications, field studies, and official investigations now confirm that the conditions for treating this waste are far from satisfactory in many facilities across the Kingdom (8, 15).

The consequences extend far beyond hospitals. The uncontrolled disposal of medical waste leads to air pollution (particularly during wild burning), soil and groundwater contamination, and increases the circulation of toxic waste in informal channels. This phenomenon primarily affects the most vulnerable populations, who are poorly informed about the associated health risks. The low level of health education, the absence of reliable epidemiological data, and the lack of specific training for hospital staff further exacerbate the problem (16, 17).

These dysfunctions call for an urgent response from health authorities. The implementation of hospital waste management policies, accompanied by adequate funding, staff training, and awareness campaigns, is essential to prevent health and environmental risks. The study aimed to assess the knowledge, attitudes, and practices (KAP) of healthcare staff regarding MPW management at El Idrissi Hospital in Kenitra, as well as their perception of the associated risks.

## Methodology

### Study design

The study was conducted at El Idrissi Hospital in Kenitra, using an anonymous questionnaire with both closed and open-ended questions. The questionnaire was structured as follows: one section concerning the respondents' personal information, another section assessing healthcare staff's knowledge of the medical record management procedure, a third section examining attitudes towards medical record management, and finally, a section dedicated to risk management.

### Population

The sample consisted of 140 respondents; 4 invalid questionnaires were excluded. The final sample size was 136 participants from various departments and services.

#### Inclusion criteria

The inclusion criteria were:

- being a member of the healthcare staff active during the data collection period;
- agreeing to participate voluntarily in the study; and
- fully completing the questionnaire.

#### Exclusion criteria

The exclusion criteria were:

- incomplete questionnaires or those containing major inconsistencies; and
- staff not directly involved in patient care (administrative staff, maintenance workers, etc.).
- A total of 140 questionnaires were distributed. After excluding four invalid questionnaires, the analysis focused on 136 respondents.

### Instrument

An anonymous questionnaire, inspired by previous work on the management of medical records and adapted to the Moroccan context, was used. It comprised four sections:

#### Sociodemographic and professional characteristics

age, sex, position, department of assignment, administrative and professional seniority.

#### Participant knowledge assessment

In this section, we assessed participants' knowledge of the medical waste management process in relation to four key elements:

- Element 1: Previous training topics (such as sorting, distribution channels, regulations, and risks associated with medical waste)
- Element 2: Methods of raising awareness about medical waste management (posters, meetings, workshops, media, or other)
- Element 3: Knowledge of medical waste categories, color codes, and bag and container fill limits
- Element 4: Knowledge of the abbreviation DASRI and national legislation regarding medical waste management.

#### Sorting compliance, knowledge level, and training

In this section, we examined the relationship between knowledge of medical waste categories and training in medical waste management using three direct questions:

- Q1: Do you have a specific code/color system for medical waste in your facility?
- Q2: Are medical waste items generated by the facility's healthcare activities systematically sorted?
- Q3: Are household waste items separated from hazardous waste at the source?

#### Strategic and organizational factors

The study of strategic and organizational factors is carried out by analyzing the interaction of the factor “existence of a waste management plan in the establishment” with the two factors: “existence of a person responsible for managing medical waste in the department (referent)” and “sufficient provision of equipment necessary for the management of medical waste”.

#### Healthcare professionals' behavior regarding the management of medical and pharmaceutical waste

To assess healthcare staff's behavior regarding the management process of pharmaceutical and medical waste, a statistical analysis was conducted to study the interaction of the factor “systematic sorting of medical and pharmaceutical waste generated during care activities” with six different elements:

- Element 1: Do healthcare staff bring bags and sharps containers to the patient's bedside?
- Element 2: Is the waste collected regularly?
- Element 3: Are waste management checks regularly carried out in your department?
- Item 4: Are the bags handled correctly (closed when two-thirds full, with gloves, not compressed, held by the top, not emptied)?
- Item 5: Are the collection personnel informed not to accept red bags and containers with sharp spikes unless they are closed?
- Item 6: Are the collected bags and containers immediately replaced with new ones?

#### Evaluation of healthcare staff attitudes

To evaluate healthcare staff attitudes regarding the management of medical records, taking into account the difficulties encountered in the department, we compared bag handling attitudes and regular monitoring of medical record management with workload, insufficient equipment and lack of training.

#### Data collection

Data collection took place over a defined period in the presence of a trained interviewer who presented the study objectives, guaranteed anonymity, and answered any questions from participants, while ensuring that their responses were not influenced. Participation was entirely voluntary, and no personally identifiable information was requested.

### Statistical analysis

The collected data were analyzed using SPSS 22. Quantitative variables were summarized using means, standard deviations, and ranges, while qualitative variables were presented as frequencies and percentages. An internal consistency check was performed before analytical processing. From an ethical standpoint, the study adhered to the principles of anonymity and informed consent and obtained prior approval from the hospital administration.

## Results

### Sociodemographic characteristics of participants

The majority of participants are women (67.6%), while 32.4% are men. Almost half of the respondents are under 35 years old (41.2%), while 36.8% are between 35 and 45 years old, and 22.1% are over 45, with a mean age of 38 years (SD = 10.4 years).

Almost half of the participants (47.1%) have less than 10 years of administrative experience, 32.4% have between 10 and 30 years, while a minority (5.9%) have more than 30 years of administrative experience, with a mean of 13 years (SD = 9.2 years) (Table 1).

**Table 1:** Characteristics of participants.

Variable		Number (%)
Gender	Female	92 (67.6)
	Male	44 (32.4)
Age (years)	<25	22 (16.2)
	25–35	34 (25.0)
	35–45	50 (36.8)
	≥ 45	30 (22.1)
	m±SD[IC95%]	37.9±10.4[36.3−39.7]
Grade	Medical doctor	22 (16.2)
	Nurse	54 (39.7)
	Health assistant	4 (2.9)
	Other	56 (41.2)
Administrative experience (years)	< 10	64 (47.1)
	10–20	44 (32.4)
	20–30	20 (14.7)
	≥ 30	8 (5.9)
	m±SD[IC95%]	12.9±9.2[11.3−14.5]

**Figure 1:**
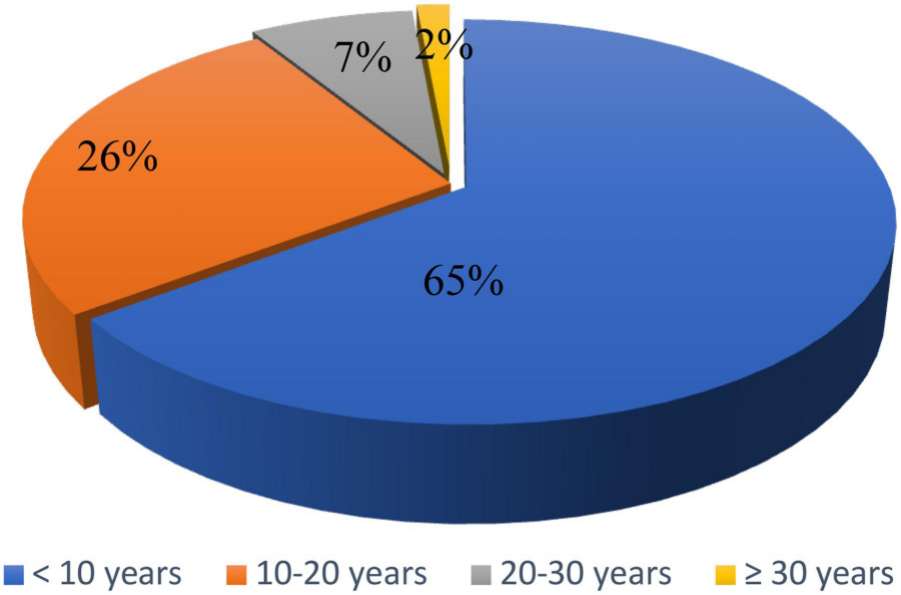
Distribution of participants according to their experience in department.

Figure 1 shows the distribution of participants according to seniority in the service, almost two-thirds of respondents have less than 10 years of seniority in the service, a little over a quarter of participants (26.5%) has between 10 and 20 years of seniority, while a minority have more than 30 years of seniority in the service.

### Knowledge assessment

The results show that the vast majority of respondents (70.6%) reported having knowledge of DMP management, while only 29.4% stated they did not have knowledge of the DMP management process (Table 2).

**Table 2:** The participants' level of knowledge on the medical waste management process.

Variable			Knowledge		Significance	
			Yes	No	Khi-2	*p*-value
Training	Sorting	Yes	60 (62.5)	2 (5.0)	37.63	***p*** **<** 0.001^***^
		No	36 (37.5)	38 (95.0)		
	Circuit	Yes	54 (56.3)	0 (0.0)	37.31	***p*** **<** 0.001***
		No	42 (43.8)	40 (100.0)		
	Regulations relating to MPWM	Yes	46 (47.9)	0 (0.0)	28.96	***p*** **<** 0.001***
		No	50 (52.1)	40 (100.0)		
	Risk related to MPW	Yes	54 (56.3)	2 (5.0)	30.61	***p*** **<** 0.001***
		No	42 (43.8)	38 (95.0)		
MPWM awareness	Displayed	Yes	70 (72.9)	4 (10.0)	45.06	***p*** **<** 0.001***
		No	26 (27.1)	36 (90.0)		
	Meeting	Yes	24 (25.0)	2 (5.0)	7.30	***p*** **=** 0.007**
		No	72 (75.0)	38 (95.0)		
	Days	Yes	18 (18.8)	0 (0.0)	8.64	***p*** **=** 0.003**
		No	78 (81.3)	40 (100.0)		
	Media	Yes	20 (20.8)	6 (15.0)	0.62	*p* = 0.431
		No	76 (79.2)	34 (85.0)		
Knowledge	MPW categories	Yes	70 (72.9)	2 (5.0)	52.77	***p*** **<** 0.001***
		No	26 (27.1)	38 (95.0)		
	Color code DASRI	Yes	70 (72.9)	0 (0.0)	60.10	***p*** **<** 0.001***
		No	26 (27.1)	40 (100.0)		
	Bag/container fill limit	Yes	44 (45.8)	2 (5.0)	21.03	***p*** **<** 0.001***
		No	52 (54.2)	38 (95.0)		
Knowledge of the abbreviation DASRI		Yes	76 (79.2)	2 (5.0)	63.50	***p*** **<** 0.001***
		No	20 (28.8)	38 (95.0)		
Knowledge of national legislation regarding MPWM		Yes	58 (60.4)	20 (50.0)	1.25	*p* = 0.263
		No	38 (39.6)	20 (50.0)		

MPWM, medical and pharmaceutical waste management.

Bold value represent significant parameter.

* *p*<0.05.

** *p*<0.01.

*** *p*<0.001.

Regarding training topics, the results show that the vast majority of respondents with knowledge of the medical waste management process (62.5%) have already received training on the sorting of pharmaceutical medical waste, with a highly significant association (*(χ*^2^ = 73.63, *p* < 0.001). Meanwhile, 56.6% of them report having received training on the medical waste circuit and risks associated with medical waste, with highly significant associations (*(χ*^2^ = 37.31, *p* < 0.001 and g^2^ = 30.61, *p* < 0.001, respectively). Finally, 47.9% of them report having received training on medical waste management regulations, with a highly significant association (*χ*^2^ = 28.96, *p* < 0.001).

The results in Table 2 show a highly significant association between knowledge of the medical device management process and the various methods of raising awareness about medical device management; namely, almost three-quarters (72.9%) of participants who reported having knowledge of the medical device management process had already received awareness training. *(χ2* *=* *45.06, p* *<* *0.001)*, A minority (18.8%) of them benefited from a day of awareness training on the management of medical waste (*χ2* = 8.64, *p* = 0.003). Meanwhile, 25% of them attended a meeting on this topic (*χ2* = 7.30, *p* = 0.007).

Conversely, we found no significant associations (*p* > 0.05) with the media as a means of raising awareness.

Statistical tests show highly significant associations between knowledge of the medical waste management procedure and certain aspects related to medical waste sorting, namely knowledge of the categories of waste produced in the hospital setting (*χ2* = 52.77, *p* < 0.001), knowledge of the infectious medical waste color code (*χ2* = 60.10, *p* < 0.001), and the fill limits of bags/containers (*χ2* = 21.03, *p* < 0.001). With 72.9% of participants possessing knowledge of medical waste management, they reported having knowledge of the different waste categories. 45.8% of these participants reported knowing the fill limits for bags/containers, while 72.9% confirmed having knowledge of the infectious medical waste color code.

Regarding the institutional aspect, the results show that 79.2% of staff familiar with the management procedure reported having knowledge of the acronym ASRI, with a highly significant association (*β*2 = 63.50, *p* < 0.001), while 60.4% of them confirmed having knowledge of national legislation on medical waste management.

### Association between knowledge, training and the practice of sorting

The results show significant associations between training in medical waste management and the three aforementioned questions (χ2 = 12.74, *p* < 0.001; χ2 = 9.42, *p* = 0.002; and gχ2 = 11.74, *p* = 0.001, respectively).

The majority (91.4%) of participants who received training in medical waste management confirmed the existence of a color-coded system for medical waste at the study site. 68.6% of them reported the systematic sorting of waste from healthcare activities within the facility, while 88.6% confirmed the separation of household waste from hazardous waste at the source (see Table 3).

**Table 3:** The association between knowledge, training and the practice of sorting.

Variable		MPWM training				Knowledge of MPW categories			
		Yes	No	Khi-2	*p*-value	Yes	No	Khi-2	*p*-value
Existence of a color/code system	Yes	64 (91.4)	44 (66.7)	12.74	***p*** **<** 0.001***	64 (88.9)	44 (68.8)	8.40	***p*** **=** 0.040*
	No	6 (8.6)	22 (33.3)			8 (11.1)	20 (31.3)		
Systematic sorting of MPW	Yes	48 (68.6)	28 (42.4)	9.42	***p*** **=** 0.002**	46 (63.9)	30 (46.9)	3.98	***p*** **=** 0.046*
	No	22 (31.4)	38 (57.6)			26 (36.1)	34 (53.1)		
Separation of household waste	Yes	62 (88.6)	42 (63.6)	11.74	***p*** **=** 0.001***	62 (68.1)	42 (65.6)	7.90	***p*** **=** 0.005**
	No	8 (11.4)	24 (36.4)			10 (13.9)	22 (34.4)		

MPWM, medical and pharmaceutical waste management.

Bold value represent significant parameter.

* *p*<0.05.

** *p*<0.01.

*** *p*<0.001.

Regarding the association between knowledge of medical waste categories and the three questions mentioned above, the results show that the vast majority of those with knowledge of waste categories reported the existence of a medical waste coding/color system in the facility (88.9%), and 68.1% confirmed the separation of household waste from hazardous waste at the source, while more than half (63.9%) confirmed the practice of systematically sorting medical waste generated by healthcare activities, with highly significant associations. (*χ2* *=* *8.40*, *p* *<* *0.04*; *χ2* *=* *7.90*, *p* *=* *0.005 et χ2* *=* *3.98*, *p* *=* *0.046 respectively*).

### Strategic and organizational factors

The majority of participants (66.2%) reported the existence of a medical record management plan, and almost three-quarters (73.3%) confirmed the existence of a person responsible for medical record management within the department, with a significant association (*β*2 = 6.08, *p* < 0.014). Furthermore, 57.8% reported having sufficient resources for medical record management (*p* > 0.05) (Table 4).

**Table 4:** Strategic and organizational factors.

Variable		Existence of a MPW management plan			
		Yes	No	Khi-2	*p*-value
Existence of a person responsible for managing MPW Availability of material	Yes	66 (73.3)	24 (52.2)	6.08	***p*** **=** 0.014*
	No	24 (26.7)	22 (47.8)		
	Yes	52 (57.8)	20 (43.5)	2.49	*p* = 0.114
	No	38 (42.2)	26 (56.5)		

Bold value represent significant parameter.

* *p*<0.05.

** *p*<0.01.

*** *p*<0.001.

### Evaluation of healthcare professionals' behavior regarding the management of medical waste

The results show that a significant majority of participants who systematically practice waste sorting confirm that healthcare staff bring sharps bags and containers to the bedside (73.7%), that MPW management is regularly monitored within the department (73.3%), and that collection staff are instructed not to collect red bags and sharps containers unless they are closed (71.1%). Furthermore, more than four-fifths report that collected containers are immediately replaced with new bags and containers (81.6%) and that waste is collected regularly (89.5%). Statistical analyses show highly significant interactions with elements 1, 2, 3, 5, and 6 (χ² = 15.70, *p* *<* *0.001, χ² = 4.06, p* *=* *0.044, χ² = 43.64, p* *<* *0.001, χ² = 19.23, p* *<* *0.001, and χ² = 10.02, p* *=* *0.002 respectively)* (Table 5).

**Table 5:** Healthcare professionals' behavior regarding the management of medical waste.

Variable		Systematic sorting of waste from healthcare activities			
		Yes	No	Khi-2	*p*-value
bring the bags and containers to the patient's bedside	Yes	56 (73.7)	24 (40.0)	15.70	***p*** **<** 0.001***
	No	20 (26.3)	36 (60.0)		
Regular collection of MPW	Yes	68 (89.5)	46 (76.7)	4.06	***p*** **=** 0.044*
	No	8 (20.5)	14 (23.3)		
Regular monitoring of MPW management	Yes	56 (73.7)	10 (16.7)	43.64	***p*** **<** 0.001***
	No	20 (26.3)	50 (83.3)		
Proper handling of bags	Yes	34 (44.7)	20 (33.3)	1.83	*p* = 0.177
	No	42 (55.3)	40 (66.7)		
Collection of bags and closed containers	Yes	54 (71.1)	20 (33.3)	19.23	***p*** **<** 0.001***
	No	22 (28.9)	40 (66.7)		
Immediate replacement of the collected bags and containers with new ones	Yes	62 (81.6)	34 (56.7)	10.02	***p*** **=** 0.002**
	No	14 (18.4)	26 (43.3)		

Bold value represent significant parameter.

* *p*<0.05.

** *p*<0.01.

*** *p*<0.001.

### Evaluation of the attitudes of healthcare professionals

The results show significant interactions between correct or incorrect bag handling and workload, insufficient equipment, and lack of training (χ2 = 17.55, *p* < 0.001, χ2 = 11.98, *p* = 0.001 and χ2 = 36.41, *p* > 0.001 respectively). However, there is a correlation between regular monitoring of DMP management and insufficient equipment (χ2 = 11.84, *p* < 0.001) and not with the workload nor with the lack of information (*p* > 0.05) (Table 6). Regarding the correct handling of waste bags, almost a third of participants handle them correctly. However, more than two-thirds (65%) fail to handle waste bags properly.

**Table 6:** Difficulties encountered by healthcare staff with regard to the management of MPW.

Variable		Proper handling of bags				Regular monitoring of MPW management			
		Yes	No	Khi-2	*p*-value	Yes	No	Khi-2	*p*-value
Workload	Yes	2 (3.7)	28 (34.1)	17.55	***p*** **<** 0.001***	18 (27.3)	12 (17.1)	2.03	*p* = 0.154
	No	52 (96.3)	54 (65.9)			48 (72.7)	58 (82.9)		
Material inadequacy	Yes	2 (3.7)	22 (26.8)	11.98	***p*** **=** 0.001***	4 (6.1)	20 (28.6)	11.84	***p*** **<** 0.001***
	No	52 (96.3)	60 (73.2)			62 (93.9)	50 (71.4)		
Lack of information	Yes	6 (11.1)	52 (63.4)	36.41	***p*** **<** 0.001***	26 (39.4)	32 (45.7)	0.555	*p* = 0.456
	No	48 (88.9)	30 (36.6)			40 (60.6)	38 (54.3)		

Bold value represent significant parameter.

* *p*<0.05.

** *p*<0.01.

*** *p*<0.001.

## Discussion

Medical waste includes all waste produced in healthcare facilities. This medical waste does not all have the same origin and does not all pose the same risk to occupational health, public health, and the environment. The current study aimed to assess the knowledge, attitudes, and practices (KAP) of healthcare staff regarding MPW management at El Idrissi Hospital in Kenitra, as well as their perception of the associated risks.

A significant portion of this waste (between 75% and 90% of the total amount of medical waste) is produced by the administrative and hospitality activities of these facilities; this waste is considered household waste and is disposed of through the regular household waste stream. However, hazardous medical waste must follow a specific disposal process, being separated from other waste from the moment of production and packaged in appropriate containers to comply with hygiene regulations and ensure the safety of individuals (patients, healthcare staff, and personnel responsible for sorting, collecting, or disposing of waste). Thus, efforts are being made to prevent accidents that may occur throughout the entire waste disposal chain (production and sorting, packaging, collection, storage, removal, transport, treatment and disposal) (3, 18).

The descriptive analysis of the participants reveals a predominantly female population (67.6%), reflecting the typical composition of staff in healthcare facilities in Morocco, where nursing and paramedical professions are largely dominated by women. This female predominance could influence perceptions and attitudes regarding the management of medical records, as several studies have shown that female healthcare workers often adopt more cautious behaviors in terms of occupational risk prevention (19–21).

Regarding age, the majority of participants were young to middle-aged: 41.2% were under 35 years old and 36.8% were between 35 and 45 years old. The mean age (37.9 ± 10.4 years) indicates a relatively experienced but still actively working population. This distribution is interesting because the literature shows that knowledge of medical record management tends to increase with experience, while practices can be improved with more frequent continuing education among younger professionals (22, 23).

The diversity of the staff represented (16.2% physicians, 39.7% nurses, 2.9% nursing assistants, and 41.2% other professionals) enriches the analysis of institutional practices. The high proportion of nurses, the main actors in the sorting and handling of medical records, is an asset for evaluating existing procedures but also makes the internal organization particularly dependent on their training and workload. The 41.2% belonging to the “Other” category suggest the varied involvement of non-clinical profiles in daily practices, which raises the question of standardization and interprofessional harmonization of procedures.

The average administrative seniority of 12.9 ± 9.2 years, with nearly half of the staff having less than 10 years of experience, shows that the team is relatively stable but still in a phase of operational learning. The low proportion of staff with over 30 years of service (5.9%) suggests likely staff turnover or structural renewal. Experience is a key factor in mastering the protocols for managing electronic health records, more health staff often have a better understanding of the risks, but not always of current standards. Seniority within the department (8.5 ± 7.3 years), lower than administrative seniority, reflects significant internal mobility. Over 64.7% of participants had been in their current department for less than 10 years. This mobility can have a dual impact: positive, by facilitating adaptation to different care environments and exposing healthcare professionals to various protocols; negative, because frequent turnover can limit in-depth mastery of EHR-specific procedures within a particular department, especially in the absence of standardized and regular training (24–26).

The distribution of experience within the department shows that nearly two-thirds of the staff (65%) have less than 10 years of service, while only 2% have more than 30 years. This high proportion of relatively new healthcare professionals may explain some variations in knowledge and practices related to medical waste management, as experience often influences the mastery of procedures.

The results show that knowledge of the medical waste management process is strongly linked to institutional awareness-raising activities, including posters, meetings, and training days, which aligns with the findings of several recent studies. Indeed, research conducted in Morocco and elsewhere has demonstrated that continuing education and the posting of procedures significantly improve knowledge levels and compliance with hospital waste sorting (8, 27). The lack of a significant association with the media as a source of awareness confirms the findings of recent research indicating that media campaigns are generally less effective than direct on-site interventions in changing healthcare practices (28). Furthermore, the highly significant association between reported knowledge and mastery of sorting (waste categories, infectious medical waste color code, fill limits) is consistent with the observations of Dunbar et al. (29), who emphasize that understanding technical standards is a major determinant of compliance (29).

The current study show that training on medical and pharmaceutical waste (MPW) management is strongly associated with improved practices, including knowledge of color coding, systematic sorting, and separation of hazardous waste, which is consistent with the findings of recent studies. Several studies conducted in North Africa and other middle-income countries have indeed shown that structured training significantly improves compliance with MPW management protocols and reduces sorting errors (30, 31). The very high proportion of trained healthcare workers reporting knowledge and application of waste sorting and separation (between 68% and 91%) confirms the positive impact of continuing education, as also reported by Bannour et al. (27), who emphasize that interactive training increases staff engagement and vigilance (27).

The significant association between knowledge of medical waste categories and reported practices (sorting, source separation, recognition of the code/color system) aligns with the observations of several researchers who found that a clear understanding of waste categories is an essential prerequisite for the correct implementation of sorting procedures (32, 33). Also, the fact that over 88% of participants familiar with medical waste categories attested to the existence of a waste coding system in their facility reinforces the findings of Abdesalam et al. (34), who indicated that the availability of a clear organizational system, combined with adequate training, is a key determinant for the safe and compliant management of hospital waste (34).

The results indicate that the majority of healthcare professionals acknowledge the existence of a medical waste management plan (66.2%) and a dedicated person in charge within their department (73.3%), which aligns with international recommendations emphasizing the importance of structured governance to ensure the safe management of hospital waste. Recent studies have indeed shown that the presence of formalized procedures and a clearly identified person in charge significantly improves compliance with sorting protocols and reduces the risks of occupational exposure (35, 36). The significant association observed between knowledge of the procedure and the existence of a person in charge confirms this organizational link. However, despite this apparent structure, only 57.8% of participants felt they had sufficient equipment, a finding frequently reported in similar studies conducted in Africa and Asia, where a lack of material resources is one of the main obstacles to the effective management of medical waste (2, 15, 37). This discrepancy between organizational framework and material availability underscores the need for logistical support to ensure the optimal implementation of medical waste management standards.

The results reveal that healthcare workers' behavior regarding the management of medical waste is generally consistent with recommended best practices, as evidenced by the high proportion of participants reporting systematic waste sorting and adherence to operational procedures. The high rates of good practices, carrying bags and containers to the bedside (73.7%), regular monitoring of medical waste management within the ward (73.3%), immediate replacement of collected containers (81.6%), and regular collection (89.5%) are consistent with the findings of recent studies highlighting that ongoing training and the presence of clear protocols significantly improve healthcare staff behavior (15, 27). The highly significant statistical association between sorting practices and several behavioral dimensions confirms that healthcare workers with good technical skills more frequently adopt compliant behaviors, which aligns with the study of Zhang et al. showing that sorting skills are a major predictor of adherence to safety measures (38). The high proportion of staff reporting that collection agents comply with container closure rules (71.1%) also aligns with the study conducted by herzing et al. (39), who emphasize the importance of inter-departmental coordination to reduce exposure risks (39). Thus, these results indicate a positive trend in behavioral practices, even though the literature reminds us that vigilance must remain constant to prevent accidents related to biomedical waste.

Healthcare staff attitudes toward the management of medical waste are strongly influenced by three key factors: workload, insufficient equipment, and lack of training. The highly significant associations observed confirm what several recent studies have highlighted: workload overload reduces healthcare workers' vigilance and increases the likelihood of improper handling of biomedical waste (4, 39). Similarly, insufficient equipment particularly the lack of suitable containers, coded bags, or personal protective equipment is recognized as a major obstacle to compliance with best practices (27, 33).These results confirm that improving staff attitudes requires an integrated approach combining increased material resources, reduced workload, and targeted ongoing training.

This study has several limitations that should be highlighted. Its cross-sectional design does not allow for the establishment of causal relationships between staff knowledge, attitudes, and practices regarding the management of electronic health records (EHRs). Since the data are self-reported, they may be influenced by social desirability bias or recall bias, especially as no direct observation of actual practices was conducted to validate participants' statements. Furthermore, the survey was carried out in a single hospital, limiting the generalizability of the results to other contexts or healthcare facilities. Finally, the lack of a qualitative approach and the failure to consider the organizational specificities of each department may have masked significant variations in EHR management.

The results of this study suggest several avenues for improving the management of medical and pharmaceutical waste:

- Ongoing staff training: Implementing regular awareness and practical training programs, tailored to each department, could reduce the gap between knowledge and practice.

Institutional policies and monitoring: Establishing an internal control system and regular reporting will ensure compliance with procedures.

- Integration of sustainable solutions: Adopting innovative technologies for the treatment and recycling of medical waste, in accordance with international environmental standards, could reduce risks to staff and the environment.
- Collaboration with health authorities: Strengthening coordination between hospitals and public health agencies to standardize protocols and disseminate best practices.

## Conclusion

The study conducted at El Idrissi Hospital in Kenitra reveals generally satisfactory levels of knowledge and practices regarding the management of medical and pharmaceutical waste, but also highlights significant gaps related to training, workload, and insufficient equipment. The significant associations observed between knowledge, behaviors, and certain organizational factors underscore the importance of continuous capacity building for staff, the availability of equipment, and the strict application of institutional procedures. While these results cannot be generalized to all healthcare facilities in the country, they constitute an important indicator of the need to improve the governance of medical and pharmaceutical waste, implement more regular audits, and promote a safety culture within hospitals. Future studies incorporating direct observation and a multicenter approach will strengthen the validity of the findings and better guide national interventions in medical and pharmaceutical waste management.

This study highlights persistent gaps in medical waste management at El Idrissi Hospital. Strengthening staff skills, improving internal organization, and regularly updating protocols are essential to ensure the safe management of hospital waste and reduce risks to healthcare workers, patients, and the environment.

## Data Availability

The raw data supporting the conclusions of this article will be made available by the authors, without undue reservation.
